# Acanthamoeba Keratitis: A Single-Institution Series of Four Cases With Literature Review

**DOI:** 10.7759/cureus.21112

**Published:** 2022-01-11

**Authors:** Clarissa Smith, Nida Ashraf, Megan Haghnegahdar, Kenneth Goins, Jessica R Newman

**Affiliations:** 1 Department of Internal Medicine, University of Kansas Medical Center, Kansas City, USA; 2 Department of Internal Medicine, Division of Infectious Diseases, University of Kansas Medical Center, Kansas City, USA; 3 Department of Ophthalmology, University of Kansas Medical Center, Kansas City, USA

**Keywords:** corrective contact lens, confocal microscopy, miltefosine, protozoal keratitis, acanthamoeba keratitis

## Abstract

*Acanthamoeba* species are free-living protozoa found pervasively in water and soil, which can cause infections of the central nervous system, skin, and eye. Amoebic keratitis (AK) is a vision-threatening, often chronic infection that is associated with the use of soft contact lenses due to corneal microtrauma and improper cleaning and storage. Although AK infections are rare, they cause significant morbidity including vision loss due to the diagnostic and therapeutic challenges they pose.

The clinical course is determined by the organism’s inherent pathogenicity, delay of diagnosis, and the paucity of data on effective therapeutic regimens. The case series and review of literature that follows examine current latest best practices in AK diagnosis including in vivo confocal microscopy (IVCM) and therapeutic interventions including miltefosine.

## Introduction

*Acanthamoeba* species are free-living protozoa found ubiquitously in soil and water and are implicated in serious infections of the central nervous system, skin, and eye [1]. Their life cycle is notable for being biphasic, with a trophozoite form capable of attaching to host epithelium and invading to deeper structures, as well as a cystic form that can endure adverse conditions including treatment attempts [1]. Human studies of serum antibodies to *Acanthamoeba* show that most individuals have been exposed to the organism; however, clinically significant infections are of relatively low incidence and frequently affect contact lens users and the immunocompromised [2,3].

Amoebic keratitis (AK) is a progressive, vision-threatening infection that is strongly associated with soft contact lenses due to contamination related to improper cleaning and storage with infested tap water. Additionally, corneal microtrauma and epithelial changes are known to occur with chronic contact use, predisposing the user to infection [4]. It is common for these infections to be chronic in nature due to the durability of the cystic form and the possibility for reinfection, which is at least partially attributed to the immunologically privileged nature of this anatomic site. Treatment is challenging due to the diagnostic delay related to initial misdiagnosis, nonspecific presentation of the disease, and the need for specialized diagnostic measures. Additionally, *Acanthamoeba* infection is oftentimes polymicrobial, including coinfections with fungi and bacteria. Without treatment of coinfections, a symbiotic relationship between the two organisms persists, causing continued damage to the cornea despite treatment of the *Acanthamoeba*. Untreated and suboptimally treated infections have been observed to spread contiguously into the deeper structures of the eye and central nervous system manifesting as granulomatous amoebic encephalitis [5]. While AK infections are relatively uncommon, they are associated with significant morbidity and vision loss due to the diagnostic and therapeutic challenges they pose.

The series of cases that follow highlight common presenting features, discuss diagnostic assays, and report on experiences of multidrug oral treatment strategies. Rapid referral to an ophthalmologist trained in confocal microscopy can be key to making an early diagnosis of AK. If topical treatments fail, systemic therapies including miltefosine, a pharmacologic agent with recent approval for refractory AK, and voriconazole may be of benefit.

## Case presentation

Case 1

A previously healthy 38-year-old female was referred to the ophthalmology clinic for three months of pruritis and photosensitivity in her left eye associated with intermittent left-sided headaches and decreased visual acuity. She failed to improve with empiric treatment for herpes simplex virus (HSV) keratitis. Exposure history was significant for use of corrective soft contact lenses. There was a history of a positive antinuclear antibody, and oral hydroxychloroquine had been started by her primary physician without improvement.

**Figure 1:**
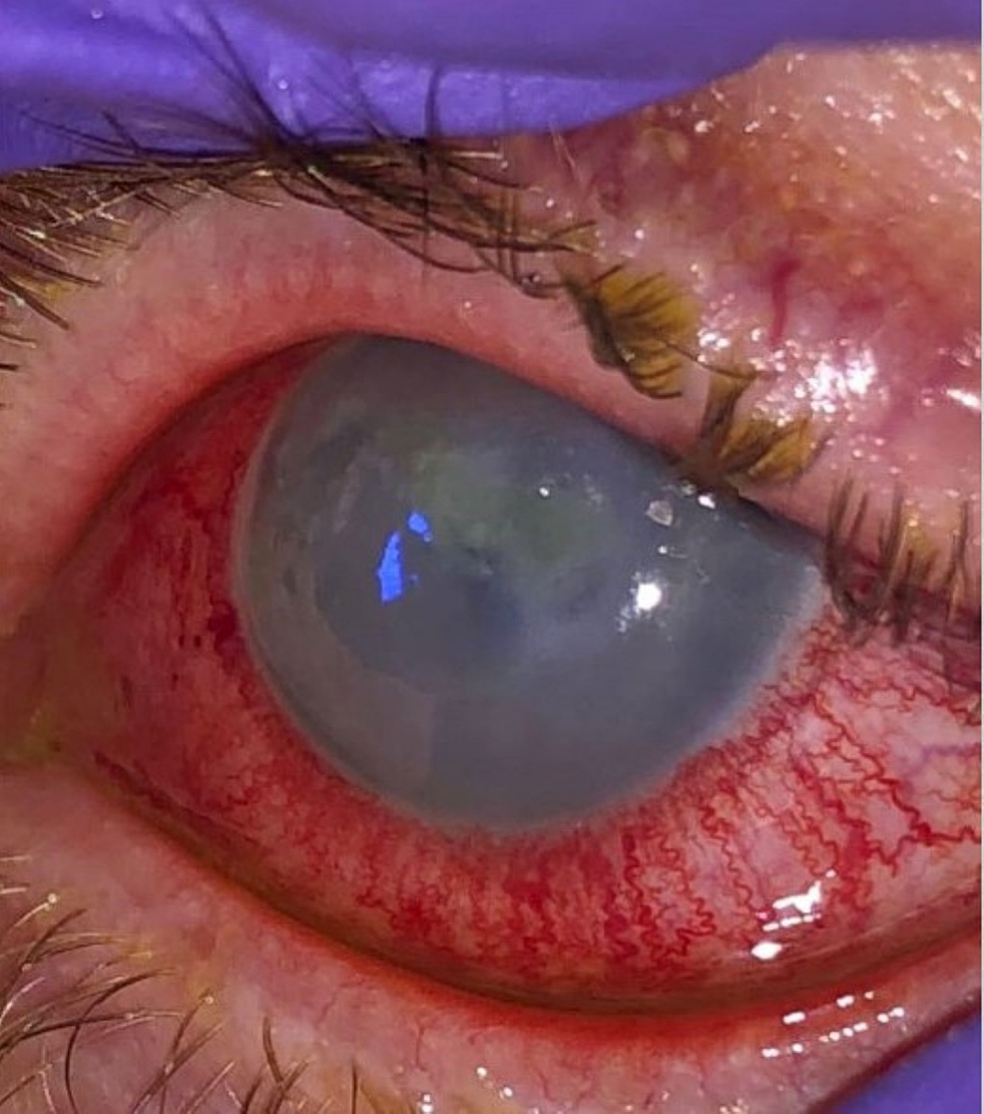
Slit-lamp examination of the left eye at presentation with diffuse conjunctival injection, central corneal epithelial defect, and diffuse corneal haze.

**Figure 2:**
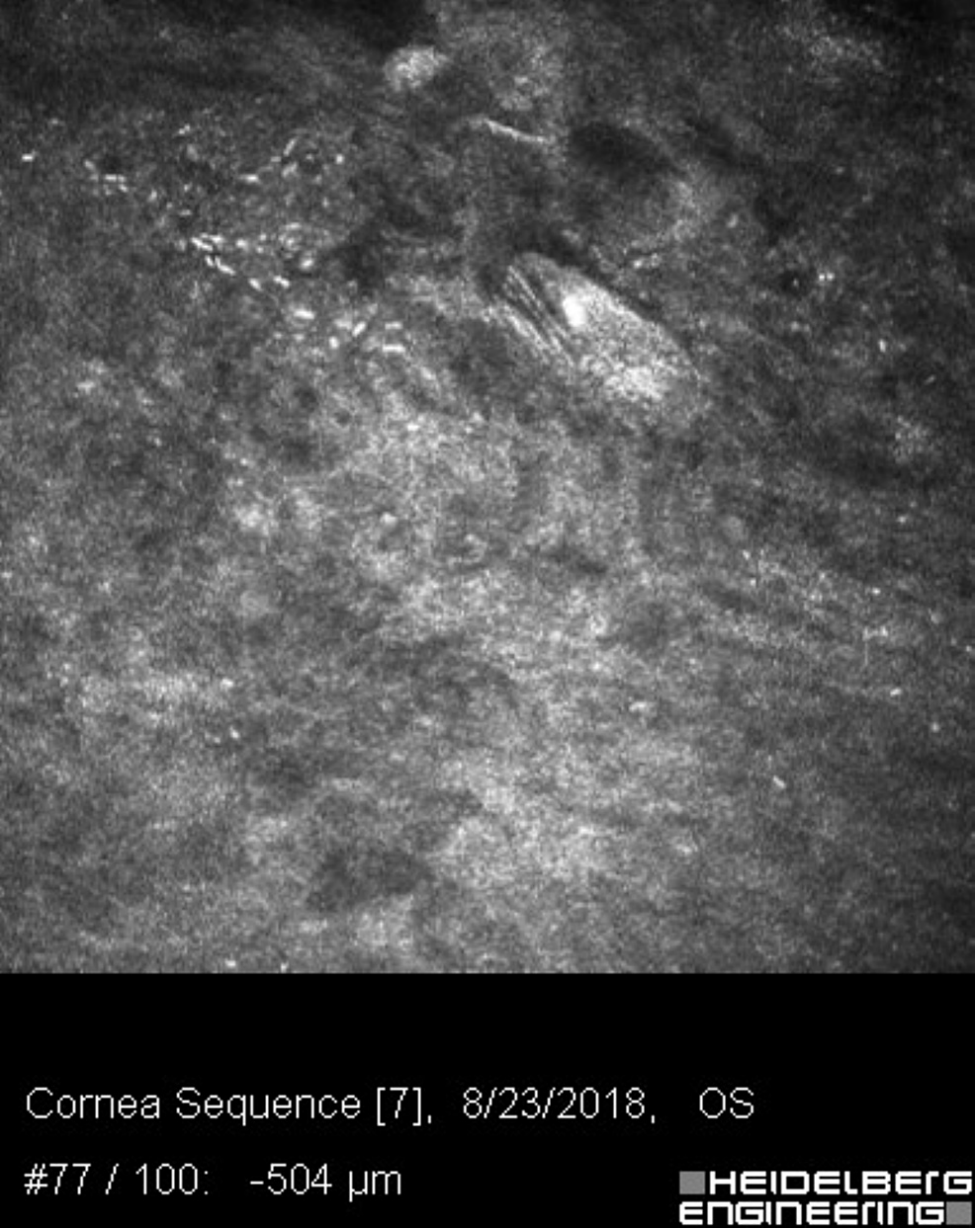
Confocal microscopy of the left cornea, 504 microns deep, with faint branching figures, consistent with either filamentous fungi or chains of bacteria (infectious crystalline keratopathy).

**Figure 3:**
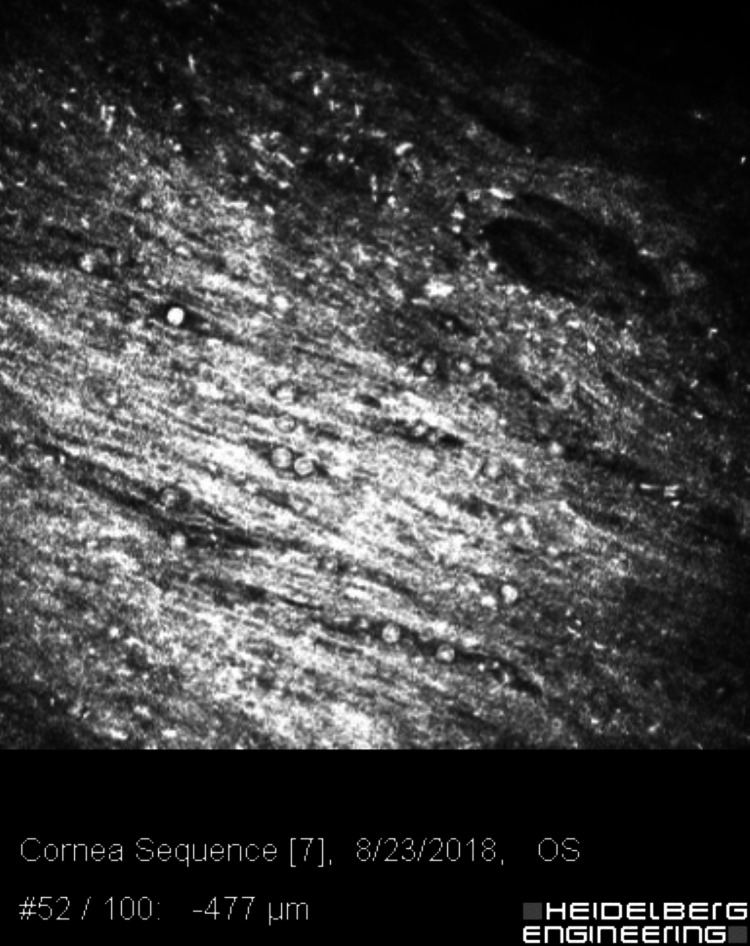
Confocal microscopy of the left cornea, 477 microns deep, showing multiple double-walled Acanthamoeba cysts.

**Figure 4:**
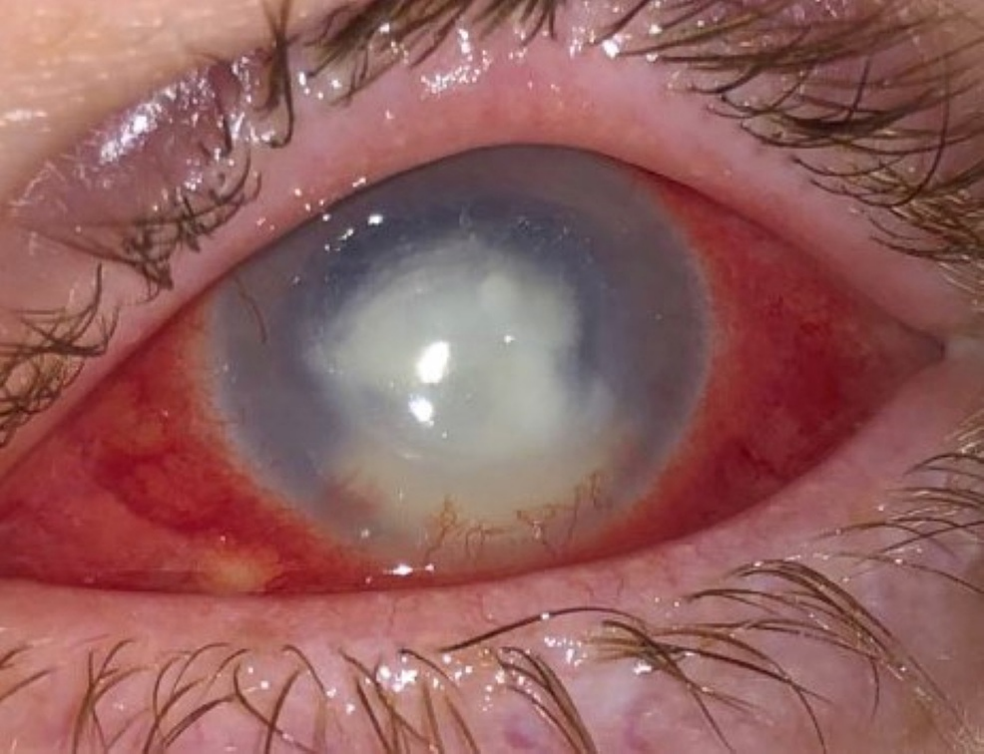
Slit-lamp examination of the left eye showing the increasing size of stromal infiltrate, anterior chamber hypopyon, and inferior scleral nodule at 7:00.

Slit-lamp examination demonstrated diffuse corneal haze, central corneal epithelial defect, and diffuse conjunctival injection (Figure 1). Confocal microscopy revealed branching hyphae (Figure 2) and multiple amoebic cysts (Figure 3). Nucleic acid amplification of corneal scrapings was positive for *Acanthamoeba*. Her condition continued to worsen, and she developed *Acanthamoeba* scleritis (Figure 4) despite topical chlorhexidine, voriconazole, moxifloxacin, and oral valacyclovir and fluconazole; therefore, she was treated with a two-week course of IV pentamidine.

**Figure 5:**
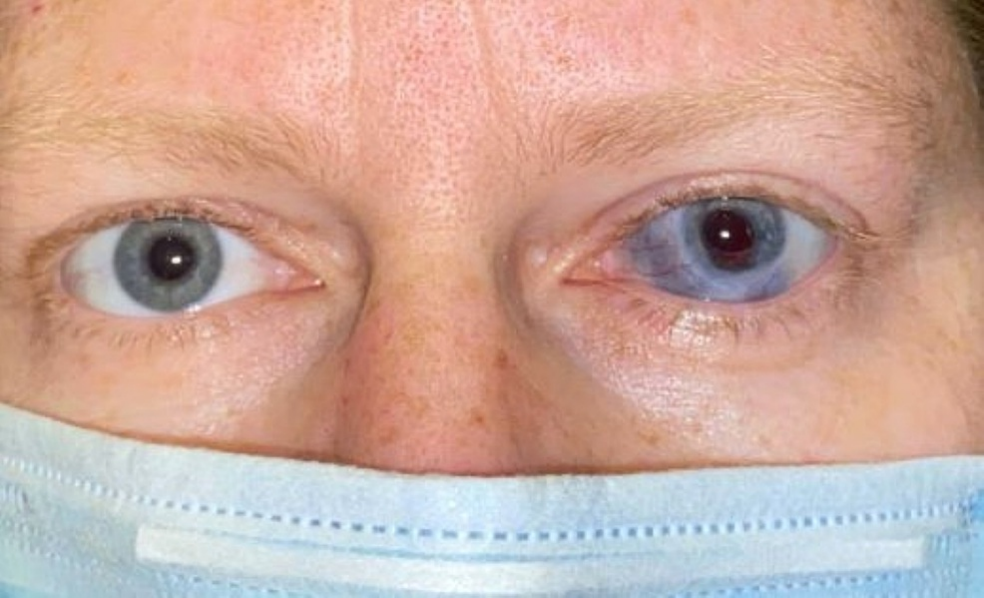
External photograph, bridge of both eyes after corneal transplantation of the left eye. There is evidence of scleromalacia (blue pigmentation of the sclera) in the left eye after resolution of Acanthamoeba sclerokeratitis. Iris heterochromia is noted due to loss of pigment related to the inflammatory process and following cataract surgery. The best-corrected vision of the left eye is 20/30 with spectacles.

With further progression of the disease, she underwent penetrating keratoplasty (PKP). Pathology of Descemet’s membrane revealed severe inflammation with numerous cysts and possible trophozoites. Post-transplantation, an aggressive regimen of oral miltefosine, voriconazole, and trimethoprim/sulfamethoxazole was added for six months. The patient continued to improve after surgery, tolerated the medications with monitoring, and remained free of disease at three years follow-up (Figure 5). Cataract surgery was required to restore best-corrected vision.

­­­­­­­­­­­­­­­­­­­­­­­­­Case 2

A 59-year-old female with an unremarkable past medical history presented to the ophthalmology clinic for an evaluation of approximately one month duration of left eye pain and photosensitivity. She had been treated previously for presumptive HSV keratitis and corneal abrasion without improvement. Symptoms progressed to include pain with extraocular movements, reduced visual acuity, and sinus pain despite appropriate topical therapy. Exposure history was significant for use of soft contact lenses, which she changed daily and denied wearing to sleep.

**Figure 6:**
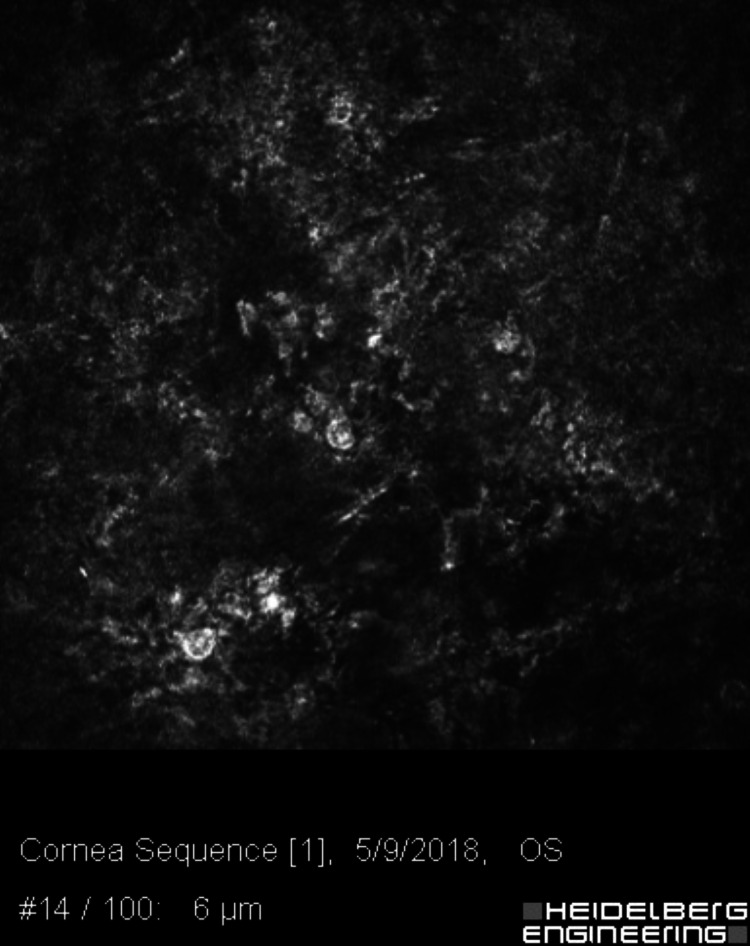
Confocal microscopy within the anterior cornea, at the level of Bowman’s layer, showing bright, round double-walled cystic structures, consistent with Acanthamoeba.

On physical examination, she had a large central epithelial defect, patchy infiltrates, satellite lesions, and a hypopyon. There was initial suspicion for fungal keratitis; therefore, she was started on oral voriconazole. Topical moxifloxacin was added, and oral acyclovir was continued. A confocal examination was performed and demonstrated bright, round double-walled cystic structures, consistent with *Acanthamoeba* (Figure 6). Topical chlorhexidine was then added. Corneal scrapings were found to be positive for *Acanthamoeba* by nucleic acid amplification.

**Figure 7:**
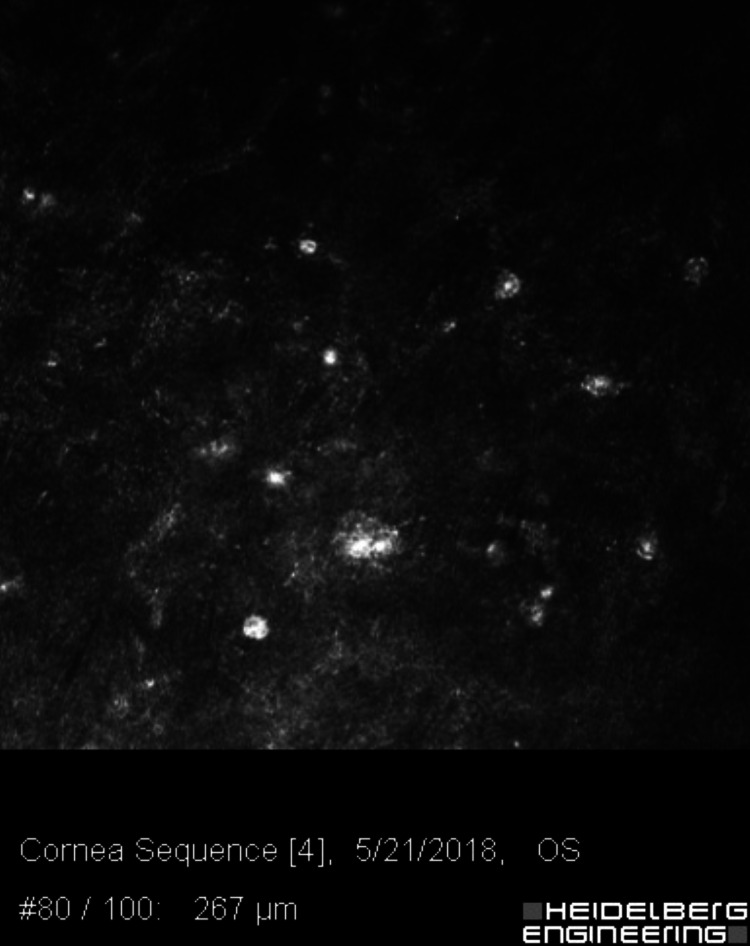
Confocal microscopy of the affected eye, 267 microns deep, in the corneal stroma showing multiple double-walled bright figures, consistent with Acanthamoeba cysts.

She continued to worsen despite topical and systemic therapy. Repeat confocal microscopy demonstrated persistence of cysts, with a deeper spread into the corneal stroma (Figure 7). The decision was made to proceed with deep anterior lamellar keratoplasty (DALK) with anterior chamber washout. Miltefosine was added to the regimen for approximately one month prior to this procedure. The corneal tissue sent to pathology was negative for *Acanthamoeba* and fungal elements. She was continued on topical chlorhexidine for an additional two weeks and no longer required oral voriconazole or miltefosine postoperatively. The patient has remained stable for three years after DALK. Cataract surgery was required to restore best-corrected visual acuity.

Case 3

A 71-year-old female with a past medical history significant for diabetes mellitus type two was referred from an outside ophthalmology clinic due to concern for worsening *Acanthamoeba* keratitis confirmed four months previously by PCR. She had been treated with propamidine isethionate and ofloxacin. She had sought care initially for an abrupt decline in visual acuity. Notably, she had worn contacts without incident for the preceding 50 years.

**Figure 8:**
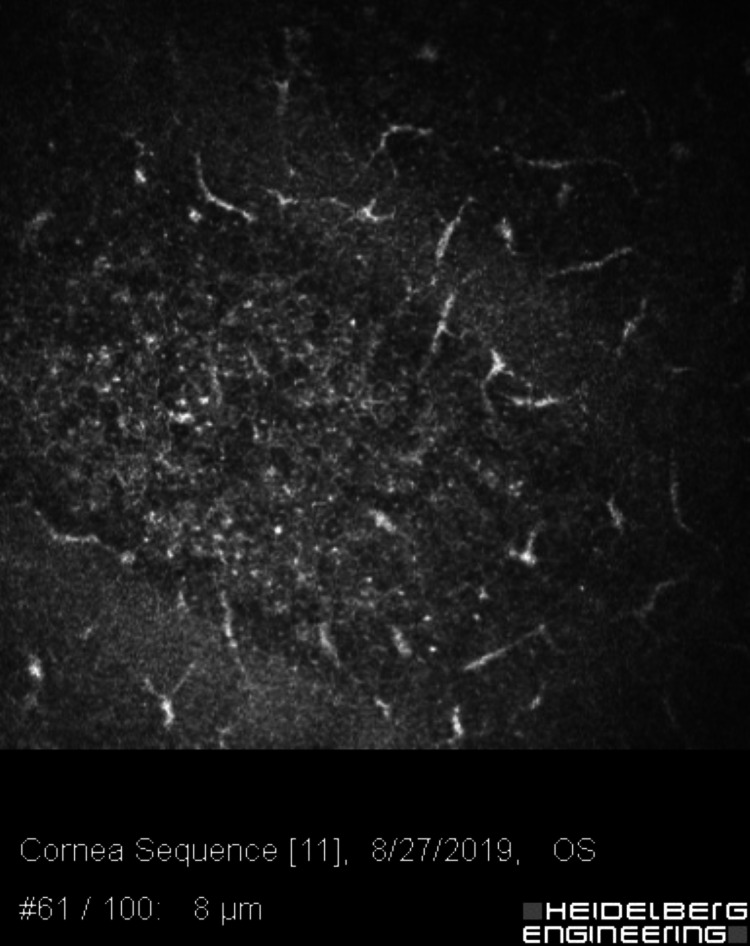
Confocal microscopy at the level between Bowman’s layer and the basal epithelium, approximately 50–70 microns deep, showing extensive Langerhans dendritic cell activation.

**Figure 9:**
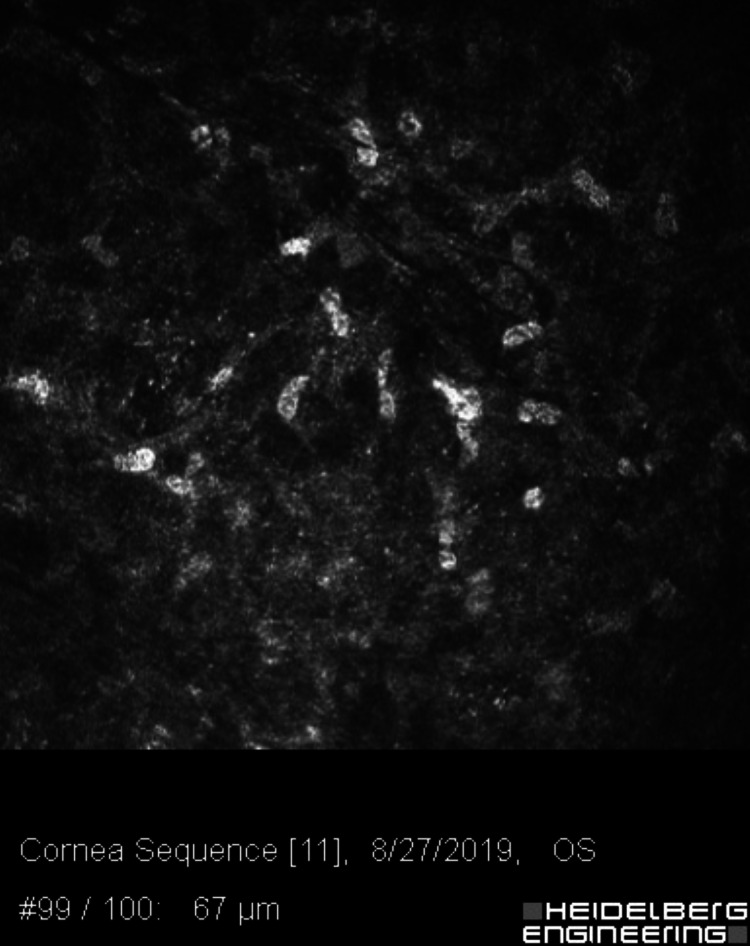
Confocal microscopy of the affected eye at the level of the anterior stroma, approximately 100 microns deep, showing multiple double-walled bright figures (Acanthamoeba cysts) in between numerous keratocytes.

Her examination showed central punctate epithelial erosions with stromal edema and haze. She was started on topical chlorhexidine and moxifloxacin. Despite treatment, her vision continued to deteriorate, and she developed eye pain, burning, and tearing. Follow-up confocal microscopic examination demonstrated numerous Langerhans cells (Figure 8), double-walled cysts (Figure 9), and extensive multilevel scarring. She continued to worsen despite treatment with topical chlorhexidine, oral voriconazole, and oral acyclovir. DALK was deemed necessary, and pathology of both anterior and deep corneal stroma demonstrated *Acanthamoeba* trophozoites. She was continued on topical chlorhexidine and oral acyclovir postoperatively.

**Figure 10:**
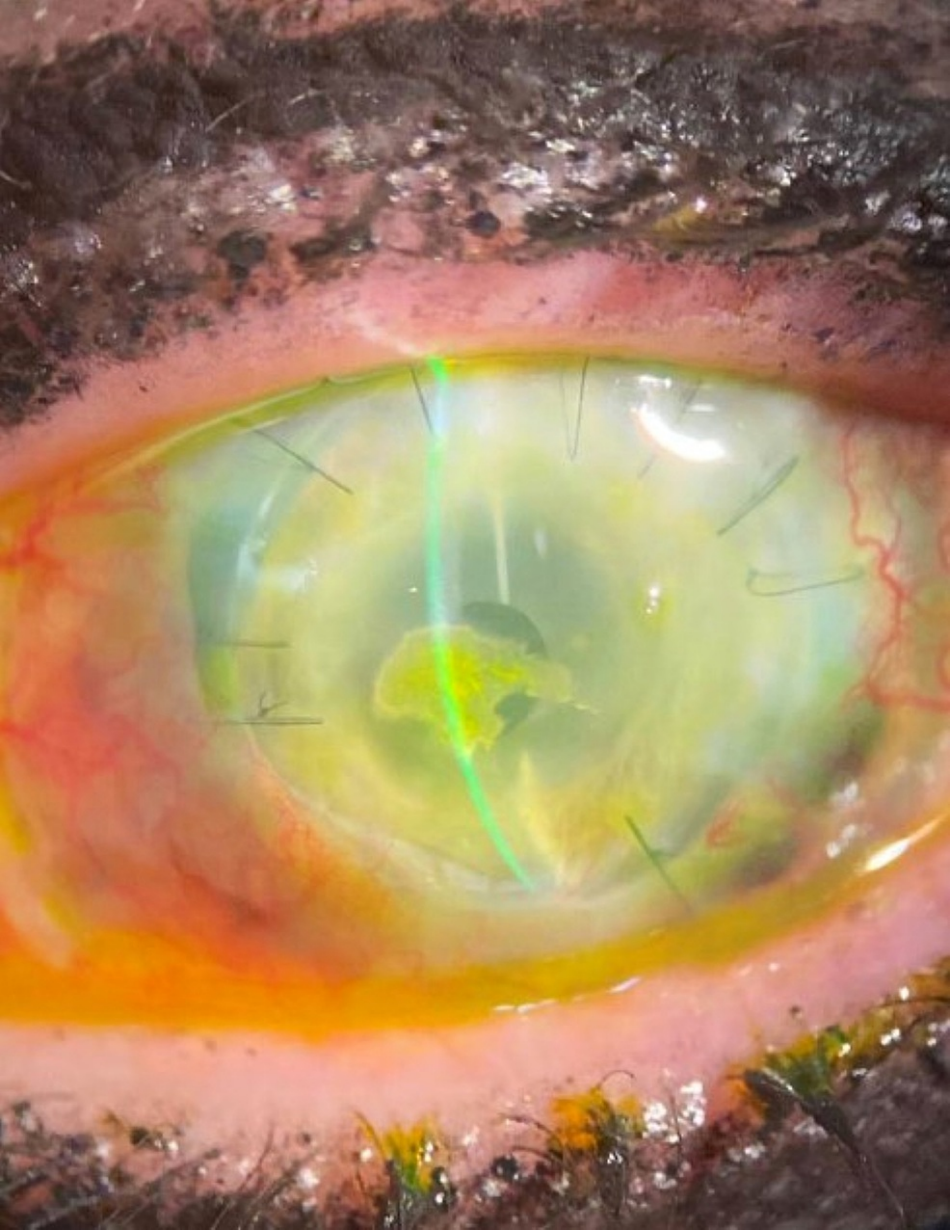
Slit-lamp photograph of left eye after a third corneal transplantation for Acanthamoeba. There is central calcific band keratopathy present and a pupillary membrane, both causing a reduction in vision.

The patient’s course was complicated by the recurrence of *Acanthamoeba* keratitis two months after DALK. New ring infiltrate and hypopyon were noted on examination, and corneal scrapings submitted for nucleic acid testing redemonstrated *Acanthamoeba* cysts. The patient was started on oral trimethoprim/sulfamethoxazole, voriconazole, and miltefosine. She was continued on topical chlorhexidine, and topical polyhexamethylene biguanide (PHMB) was added. Due to rapid progression, urgent penetrating keratoplasty was pursued with persistent *Acanthamoeba* cysts noted on pathology. The patient had tolerability problems with the miltefosine; therefore, this was discontinued. She was continued on oral voriconazole, trimethoprim/sulfamethoxazole, and topical PHMB until the disease appeared to be in remission. She then underwent cataract surgery to restore vision. Unfortunately, she developed corneal allograft failure necessitating penetrating keratoplasty again. Pathology was negative for protozoa; therefore, PHMB was discontinued. She currently has a guarded long-term prognosis due to chronic *Acanthamoeba* keratouveitis secondary glaucoma and neurotrophic keratopathy (Figure 10).

Case 4

A 46-year-old female with a history of eosinophilic esophagitis, positive antinuclear antibody, and trigeminal neuralgia was referred from an outside ophthalmologist due to approximately five months of recurrent right eye burning, tearing, vision changes, photophobia, and unilateral headache. Her symptoms appeared shortly after she began storing her contact lenses in tap water. She was initially treated by an outside optometrist for presumed herpetic keratitis with minimal relief.

**Figure 11:**
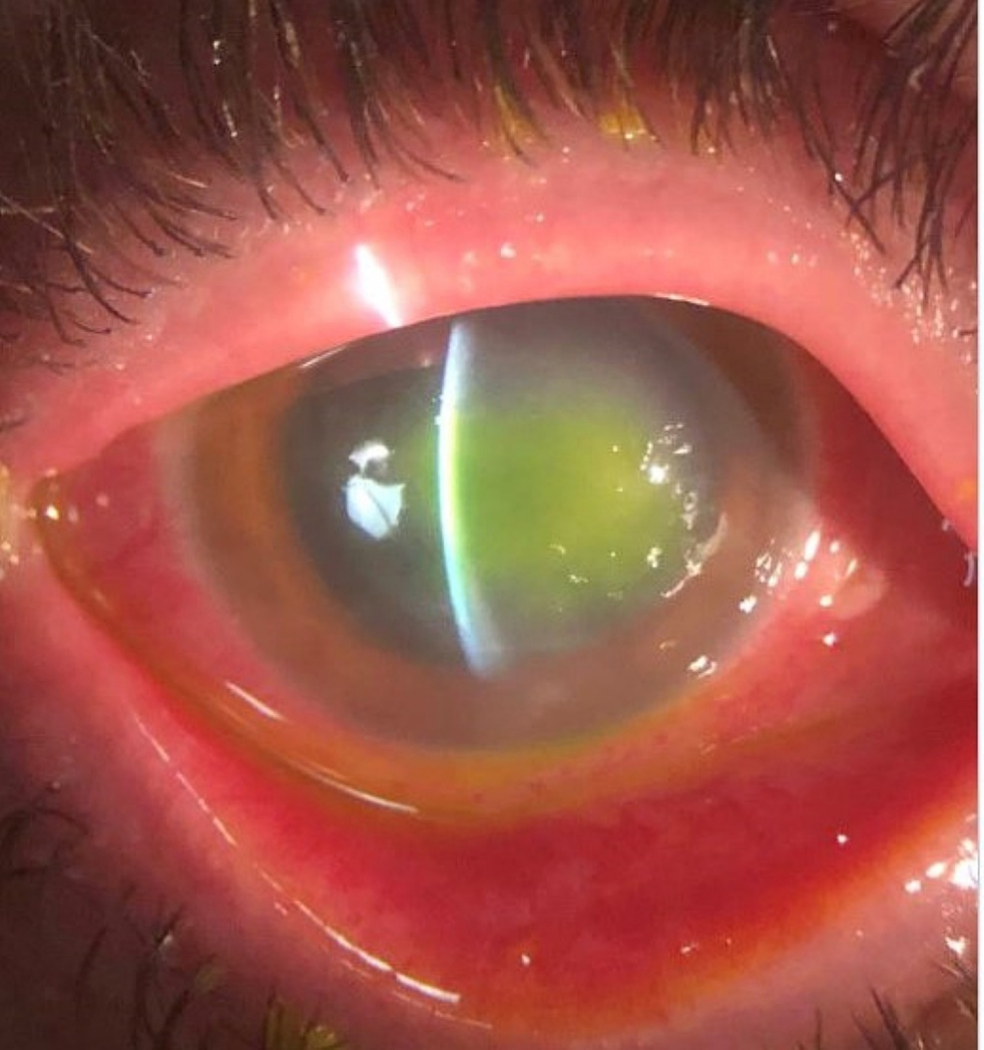
Slit-lamp examination showing a central geographic ulcer with ring infiltrate.

**Figure 12:**
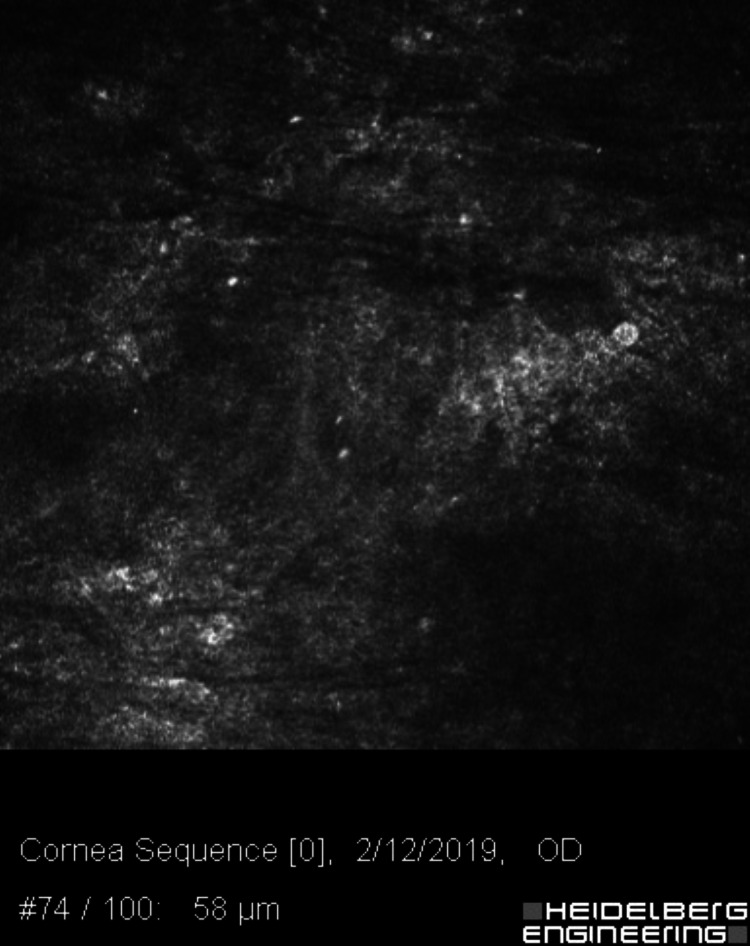
Confocal microscopy at the level of Bowman’s layer, approximately 60 microns deep, showing numerous bright figures with halos and an occasional signet ring cell, both consistent with Acanthamoeba cysts.

**Figure 13:**
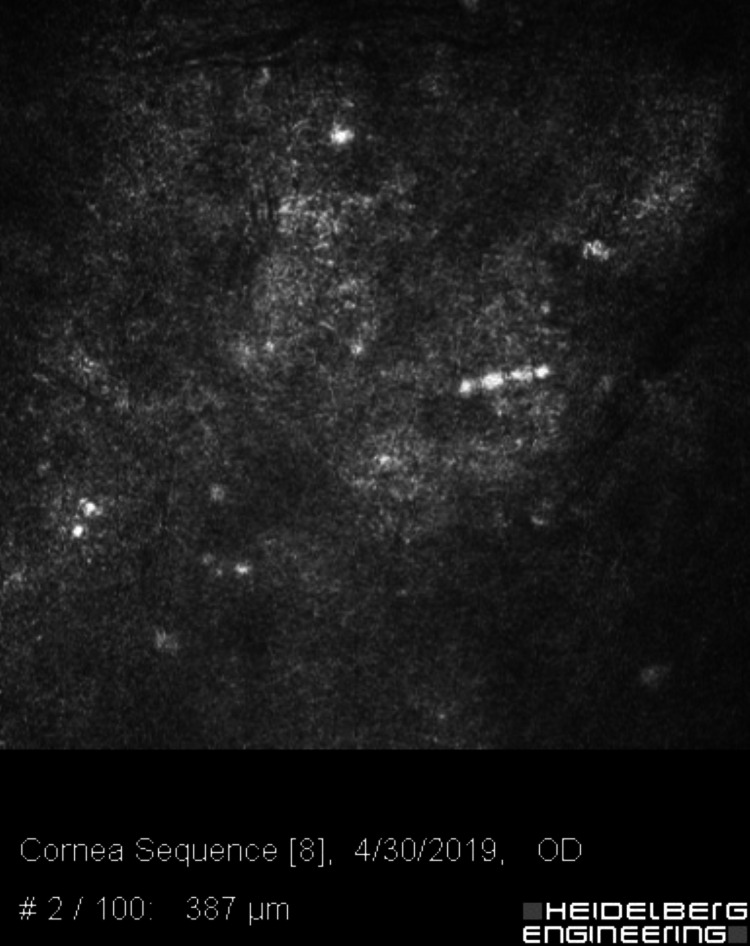
Confocal microscopy of the affected eye, 387 microns deep in the corneal stroma, showing bright figures with halos and an occasional double-walled structure consistent with Acanthamoeba cysts.

Her examination was notable for the presence of a ring infiltrate and geographic ulcer of the right cornea (Figure 11). A confocal examination demonstrated cystic amoeba forms in the corneal stroma strongly suspicious for *Acanthamoeba* (Figure 12); however, the scrapings sent for culture grew only *Pseudomonas aeruginosa*. She was started on topical PHMB, vancomycin, tobramycin, voriconazole, and oral valacyclovir. Due to incomplete resolution and strong clinical suspicion for *Acanthamoeba*, oral trimethoprim/sulfamethoxazole, voriconazole, and miltefosine were added to the therapeutic regimen. Repeat confocal microscopy demonstrated attenuated organisms near the surface and deep double-walled cysts (Figure 13). Penetrating keratoplasty was performed, and pathology demonstrated *Acanthamoeba* trophozoites, with some “empty” cysts and severe reactive changes of the corneal epithelium.

**Figure 14:**
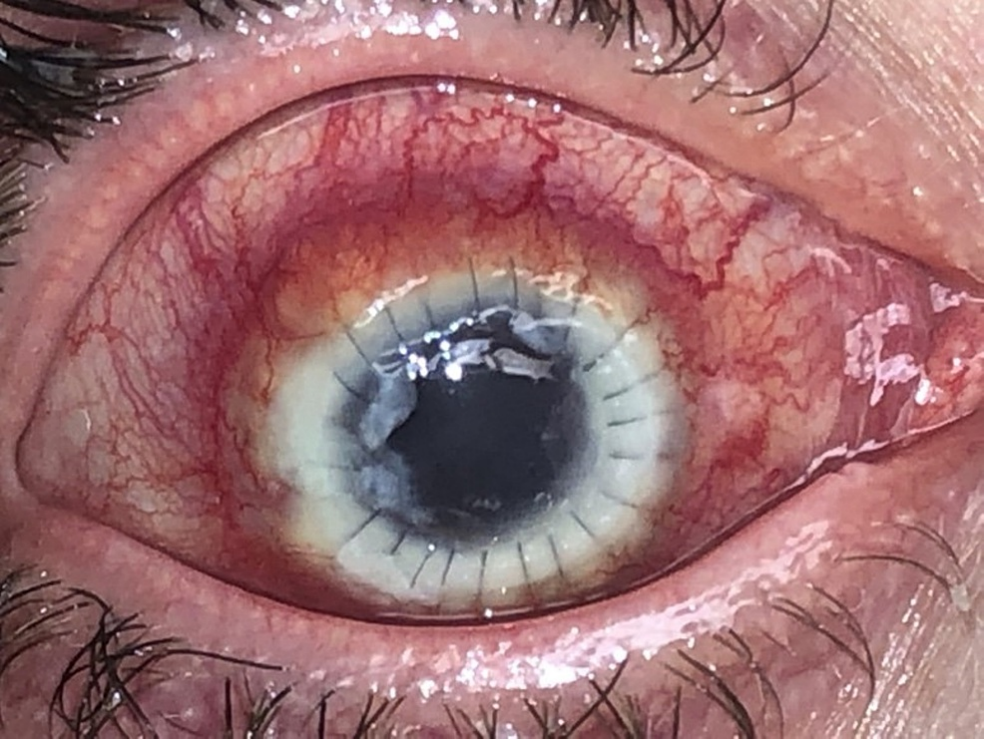
Slit-lamp photograph of the right eye with clear central corneal transplantation but new 360-degree limbal infiltrate with extension into the sclera secondary to persistence of Acanthamoeba infection.

**Figure 15:**
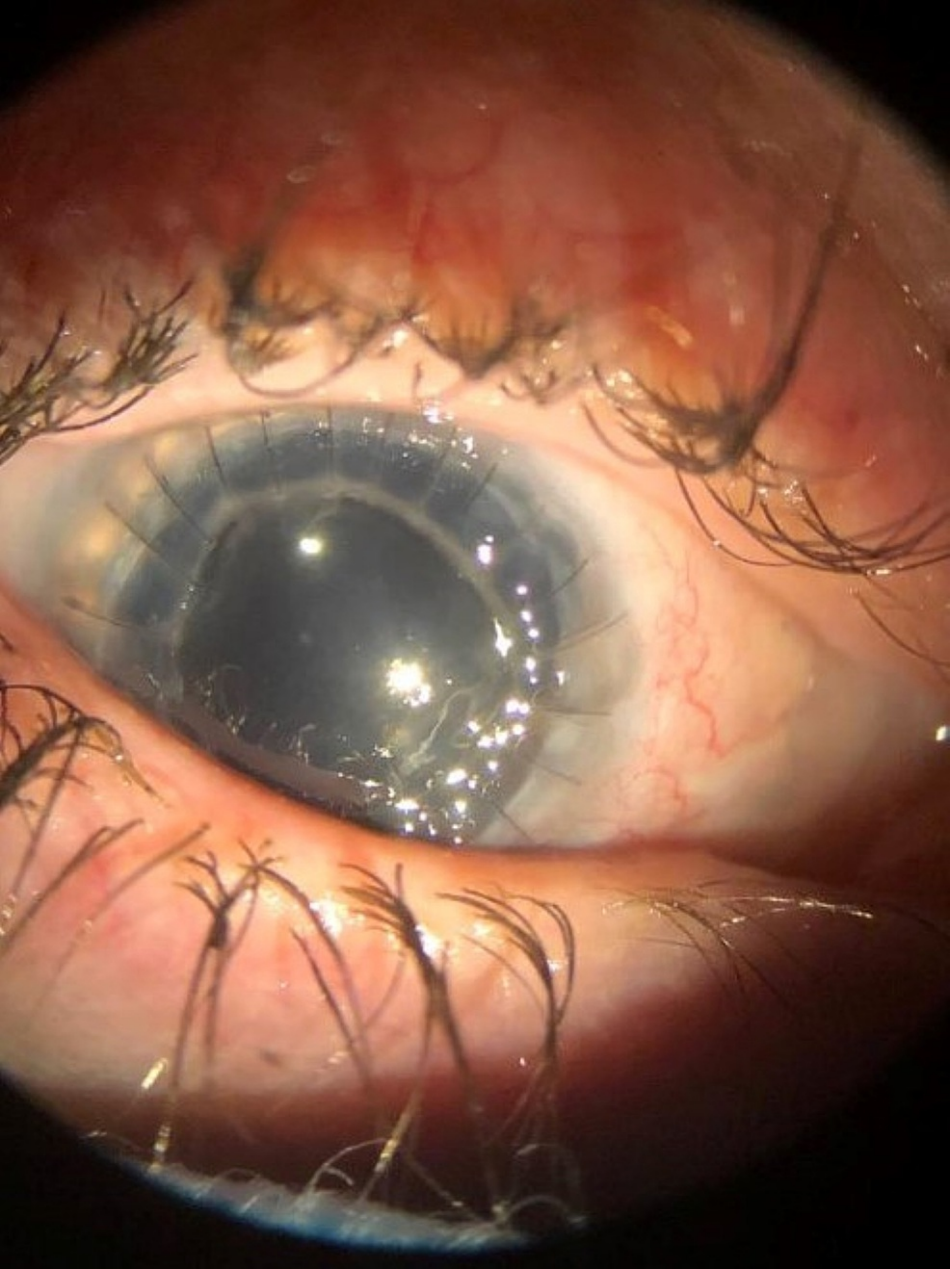
Slit-lamp examination six months after penetrating keratoplasty inside of sclerokeratoplasty. The best-corrected vision is 20/800 in relation to cystoid macular edema.

This patient’s postoperative course was complicated by disease persistence with progression involving the native sclera and uvea (Figure 14). Corneal transplantation via sclerokeratoplasty was performed and was again complicated by recurrent infection of the grafted tissue. However, failure of the sclerokeratoplasty was felt to be due to inflammation from *Acanthamoeba* keratouveitis, a response to dead or attenuated organisms in the corneal stroma. After the inflammation was controlled with oral mycophenolate mofetil, a third penetrating keratoplasty with cataract surgery was done to restore vision. Due to intolerances of trimethoprim/sulfamethoxazole and azole antifungals, she was continued on oral miltefosine with flucytosine for six months after transplantation with no evidence of persistent or recurrent disease (Figure 15). Her postoperative course was complicated by neurotrophic keratopathy requiring supraorbital nerve transposition to the right cornea and tarsorrhaphy.

## Discussion

Amoebic keratitis is an important diagnosis to consider in the presentation of clinical keratitis due to its ability to cause profound loss of vision and potential to cause life-threatening disease through invasion into the central nervous system. Its morbidity may be attributed to a variety of factors, some intrinsic to the pathogen and some ecological. The *Acanthamoeba* life cycle is inherently virulent due to the ability of the cystic form to survive inhospitable environments, including treatments that would otherwise be efficacious [1]. The trophozoite form thrives using the epithelium of the eye as an ideal portal of entry due to the concentration of mannosylated glycoproteins, which is further upregulated by the microtrauma caused by contact lens use [1,3,4].

Maintaining contact lenses also enhances the probability of direct inoculation through activities such as handling, cleaning, and storing the lenses with contaminated hands or storage products. Estimates suggest that 85% of cases are associated with the use of contact lenses, although studies have been inconclusive as to whether extended wear lenses or disposable daily lenses have higher risk [3]. Infections are not limited to contact lens users, and one study suggests that those infections may behave more aggressively [3]. Activities such as swimming, bathing, and outdoor work can introduce amoeba to the ocular structures in these individuals as well.

Another reason for this pathogen’s high morbidity is delay of diagnosis. As demonstrated in the cases above, this may be due to the overlap of symptoms and co-occurrence with other more common etiologies such as trauma, viruses (particularly herpes simplex virus), and bacteria [5]. A further complicating factor is that *Acanthamoeba* species have a well-documented propensity for symbiosis with fungal and bacterial species; therefore, multi-organismal infections should not be excluded [4,5].

Diagnosis is complicated by various limitations of diagnostic testing. Although culture remains the gold standard (with 50%-75% sensitivity and 100% specificity), multiple recent studies have demonstrated its significantly inferior sensitivity compared to that of in vivo confocal microscopy (>90% sensitivity and specificity) [6,7]. An added benefit of confocal microscopy is that it offers an immediate diagnosis, allowing for rapid initiation of treatment. The caveat is that it requires expensive equipment and a trained specialist and is not readily available in most eye clinics. If a specialist is not readily available, this may result in delay of diagnosis and treatment [5]. Much like culture, PCR relies heavily on specimen collection technique and yield, which requires invasive procurement of specimen, although PCR has improved sensitivity (71.4%-84%) and specificity (100%) [8,9]. Finally, direct cytological evaluation of corneal scrapings has sensitivity and specificity comparable to PCR and culture but still requires an invasive procurement procedure. Even minor delays in diagnosis may have associated morbidity because protozoa can invade deeper into contiguous tissues and transition into more durable and treatment-resistant cystic forms. Therefore, the goal is to initiate treatment as early and aggressively as reasonable.

Optimizing treatment of AK is an active area of investigation. The most widely accepted first-line treatments include a combination of two or three of the following topical therapies aimed at disrupting organism membranes: 0.1% propamidine isethionate, 0.02%-0.04% chlorhexidine, and 0.02% polyhexamethylene biguanide (PHMB) [10]. More concentrated doses of PHMB have demonstrated superior efficacy in locally advanced cases [10]. Greatest efficacy has been shown when doses are “pulsed” in a way that encourages excystment to the more susceptible trophozoite phase [10].

Miltefosine is a recently approved option for treatment-resistant AK with activity against both the trophozoite and cystic forms, which has shown promise in otherwise refractory cases [11]. Notably, it was originally developed in the 1980s as an antineoplastic agent; however, levels required to be sufficiently antineoplastic would be nearly universally lethal [12]. Its mechanism of action is not fully understood; however, it mimics the structure of membrane phospholipids with notable omission of glycerol. This provides the ability to disrupt phospholipid-rich membranes and cytochrome c, inducing apoptosis [12]. This potency was successfully harnessed in early 2017 for leishmaniasis, as the protozoal membranes were found to be even more susceptible than human cells. Because cellular and organelle membranes are a ubiquitous target, this medication is not without risk for significant off-target toxicity, including teratogenicity, nausea, vomiting, fever, headache, and nephrotoxicity. Less frequently, it can have severe outcomes, such as Stevens-Johnson syndrome and profound thrombocytopenia [13]. Laboratory and animal studies have yielded promising results; however, large-scale studies of efficacy in humans are lacking due in part to the rarity of the condition. Numerous case studies have remarked on its anecdotal efficacy by measure of final visual acuity, avoidance of surgery, and decreased time to cure [14,15].

The role of voriconazole also remains uncertain, although anecdotally it has shown compelling benefit. When administered topically, it is of very low risk with the main intolerance being discomfort. The contentious nature of systemic use arises from the relative paucity of in vivo data. One in vitro study suggested that voriconazole could have an antagonistic effect when coadministered with cysticidal chlorhexidine and propamidine [16]. However, another in vitro study speculated that voriconazole should be effective through the inhibition of ergosterol synthesis via 14α-demethylase [17]. Oral voriconazole has also been shown to be curative as monotherapy in two published cases, and other case studies have suggested systemic efficacy in combination with topical agents [18].

The role of steroids in the treatment of AK is often disputed. It increases the pathogenicity of the organism by enhancing excystment whether given intravenously or topically. Although increasing the burden of the active trophozoite form certainly bears the possibility of worsening the infection, it also increases the organisms’ overall susceptibility to antiamoebic therapy [3]. Steroids also quell the inflammatory response to the organism, which is responsible for some of the disease morbidity [1].

Because of well-documented coinfections and known synergism between the protozoa and certain bacteria, some therapeutic regimens include an antibacterial agent, such as topical moxifloxacin, ciprofloxacin, trimethoprim-sulfamethoxazole, or erythromycin [6]. These are of unproven efficacy but may be useful empirically or perioperatively and are generally well tolerated.

Medical management is generally attempted prior to surgery unless there is immediate threat to central nervous system penetration. Procedures such as penetrating keratoplasty (PKP) and deep anterior lamellar keratoplasty (DALK), which were originally performed with the intention of debriding diseased tissue and debulking disease, are now generally reserved for either cases refractory to medical management or for restoration of vision due to structural damage [6,19,20].

## Conclusions

In conclusion, it is essential to include AK on the differential diagnosis for patients with keratitis, especially, but not exclusively, in those who wear contact lenses. Should a patient on empiric topical/oral therapies develop a further decline in vision, worsened eye pain, photophobia, or increased injection, they should communicate this promptly to their care team and be seen urgently for a repeat examination. Additionally, AK cannot be excluded in patients proven to have a viral, bacterial, or fungal infection, as coinfection is relatively common. Similarly, AK should not be excluded if there is high clinical suspicion, even if culture or PCR is negative due to poor sensitivity.

Rapid referral to an ophthalmologist trained in confocal microscopy can be key to a timely diagnosis and therefore improved outcome. In cases refractory to standard topical treatments, systemic therapies including miltefosine and voriconazole may be of benefit. Surgery should generally be reserved for central nervous system penetration or visual acuity restoration.

## Appendices

Table 1 shows the summary of the cases.

**Table 1:** Summary of the cases IVCM: in vivo confocal microscopy

	Case 1	Case 2	Case 3	Case 4	Literature review
Age	38	59	71	46	Median: 31 ± 13 [20]
Risk factors	Soft contact lens use	Soft contact lens use	Soft contact lens use, recent AK infection	Soft contact lens use	Soft contact lens use (85%) [3], trauma (40% of non-contact lens cases), exposure to contaminated water including pools and hot tubs and soil sources [3]
Symptoms	Unilateral pruritis and photosensitivity	Unilateral photosensitivity	Decreased visual acuity	Unilateral photosensitivity, tearing, decreased visual acuity	Pain (95.3%), photophobia (37.2%), foreign body sensation (23%) [20]
Time from symptom onset to diagnosis	2.5–3 months	2 weeks	2 months	4.5 months	Median: 2 (range: 0–26 weeks) [20]
Examination	Geographic corneal ulceration	Conjunctival injection with hypopyon	Ring infiltrate and hypopyon	Ring infiltrate and geographic corneal ulcer	Stromal infiltrates (68.2%), advanced AK signs such as ring infiltrate, stromal impairment, hypopyon (68.2%) [20]
Diagnosis	IVCM, confirmed by PCR	IVCM, confirmed by PCR	PCR at OSH, IVCM and PCR to confirm recurrence	IVCM with cysts, but PCR was negative, and culture with only *Pseudomonas aeruginosa*	Culture, cytology of scrapings IVCM, PCR
Treatment	First line: topical chlorhexidine, voriconazole, moxifloxacin, and atropine, as well as oral valacyclovir and fluconazole; second line: IV pentamidine; miltefosine, Bactrim, and voriconazole postoperatively added	First line: acyclovir; second line: topical chlorhexidine, moxifloxacin, and voriconazole; third line: preoperative miltefosine; voriconazole postoperatively added	First line: topical chlorhexidine, ofloxacin, and oral acyclovir; second line: Bactrim DS, voriconazole, and miltefosine and topical PHMB	First line: topical PHMB, voriconazole, moxifloxacin, and erythromycin; second line: oral Bactrim DS, voriconazole, and miltefosine	First line: topical biguanides and diamidines; refractory cases: oral miltefosine; unproven benefit: voriconazole, steroids, and antibacterial, antifungal, antiviral agents
Surgical intervention	PKP	DALK	DALK, PKP	PKP, sclerokeratoplasty	Reserved for invasive, refractory, or posttreatment sight restoration
Pathology	Numerous cysts and possible trophozoites	No protozoa	Anterior and deep corneal stroma demonstrated trophozoites	Trophozoites, cysts some “empty” cysts	Frequently shows encysted amoebic forms and chronic reactive changes, bacteria, and fungal structures [20]
Outcome	Disease-free, confirmed by IVCM	Disease-free, confirmed by IVCM	After multiple relapses, disease-free with ongoing close follow-up	After relapses, requiring two additional debridement procedures and grafts, the patient is now disease-free	Recurrence is common; vision improved from presentation in 63.9%, unchanged in 16.7%, and deteriorated in 19.4% [20]
Postinfection Snellen acuity in the affected eye	20/60	20/150	20/200	Hand movement	Variable
